# Long-term brain health among females with new-onset hypertensive disorders of pregnancy

**DOI:** 10.1016/j.xagr.2026.100704

**Published:** 2026-10-07

**Authors:** Amanpreet Kaur, Adriana Angarita Fonseca, Hassan Behlouli, M. Natasha Rajah, Louise Pilote

**Affiliations:** 1 Department of Medicine, Faculty of Medicine and Health Sciences, McGill University Health Centre, Montreal, Canada (Kaur and Pilote); 2 Centre for Outcomes Research and Evaluation, Research Institute of the McGill University Health Centre, Montreal, Canada (Kaur, Fonseca, Behlouli, and Pilote); 3 Department of Psychology, Faculty of Arts, Toronto Metropolitan University (Rajah); 4 Department of Psychiatry, Faculty of Medicine and Health Sciences, McGill University, Montreal, Canada (Rajah)

**Keywords:** brain structure, cerebral small vessel disease, cognitive function, gestational hypertension, gray matter volume, magnetic resonance imaging, white matter hyperintensities, white matter microstructure

## Abstract

**BACKGROUND:**

Hypertensive disorders of pregnancy are key female-specific risk factors for vascular diseases. Given the association between vascular health and brain structure and function, it remains unclear whether new-onset hypertensive disorders of pregnancy is associated with later-life brain changes and cognition.

**OBJECTIVE:**

Our objective was to evaluate the association between a history of hypertensive disorders of pregnancy and brain structural changes and cognition in middle-aged and older females using data available from the UK Biobank cohort.

**STUDY DESIGN:**

We conducted a matched case–control analysis using data from the UK Biobank. We compared females with a history of new-onset hypertensive disorders of pregnancy to females with normotensive pregnancies, matched 1:4 (case:control) on age at delivery and number of births. We included females with available brain magnetic resonance imaging and cognitive assessments (mean age ∼30 years postpartum). We used linear regression models, adjusting for known clinical and demographic covariates, to assess brain volumes, white matter hyperintensities, white matter microstructural integrity, and a neurocognitive battery of tests.

**RESULTS:**

When compared with females with a history of normotensive pregnancy, females with a history of hypertensive disorders of pregnancy had lower gray matter volume (β=−6.0 mL; 95% confidence interval, −10.9 to −1.1; *P*=.02), as well as an increase in the volume of white matter hyperintensities (β=0.5 mL; 95% confidence interval, 0.1–0.9; *P*=.02). No significant differences were observed in white matter microstructural integrity or in neurocognitive tests.

**CONCLUSION:**

A history of hypertensive disorders of pregnancy is associated with later-life differences in brain structure decades after pregnancy, with lower brain volumes and a higher white matter hyperintensities burden. These findings suggest that hypertension during pregnancy may present an early life vascular exposure with long-term subclinical consequences for brain health.

**AJOG Global Reports at a Glance**

**Why was this study conducted?**

Hypertensive disorders of pregnancy (HDP) are female-specific vascular risk factors, but their long-term impact on brain structure and cognition remains unclear.

**Key findings**

Females with a history of HDP had lower gray matter volume and a higher volume of white matter hyperintensities decades after pregnancy.

No significant differences were observed in cognitive performance or white matter microstructural integrity.

**What does this add to what is known?**

HDP may represent a sex-specific risk factor for compromised brain health not only in the immediate postpartum period but also years later in life.

## Introduction

There is growing evidence that sex differences exist in dementia risk; for example, females account for 66% of late-onset Alzheimer disease (AD) cases.^1^ This disproportionate prevalence in females is multifactorial.^2^ Although longer life expectancy in females is a significant contributor, it does not fully explain this disparity.^3^^,^^4^ Therefore, it is important to understand sex-specific biological and life-course exposures, such as pregnancy, and how these experiences may interact with modifiable vascular risk factors for dementia, potentially placing some females at an increased risk for adverse brain health later in life.^5^^,^^6^

New-onset hypertensive disorders of pregnancy (new-onset HDP), which include gestational hypertension and preeclampsia, are key female-specific risk factors for vascular diseases. New-onset HDP are major risk factors for maternal and neonatal morbidity and mortality.^7^^,^^8^ The effects of gestational hypertension—that is, high blood pressure (systolic ≥140 or diastolic ≥90 mm Hg) developed during the 2^nd^ or 3^rd^ trimester^8^^,^^9^—include maternal nephropathy, low birth weight, preterm delivery, and, in more serious cases, preeclampsia.

New-onset HDP is associated with cognitive impairment during the immediate postpartum period. However, the adverse effects of new-onset HDP on cognitive function later in life remain less clear.^8^^,^^10^^,^^11^ New-onset HDP can affect cognitive function either by sharing common risk factors, including obesity, diabetes, hypertension, and hyperlipidemia^11^; and/or through endothelial damage sustained during new-onset HDP, particularly preeclampsia. Indeed, damage to large vessels as well as the microvasculature may persist years after an affected pregnancy.^12^^,^^13^ These subclinical changes may, therefore, affect the vascular bed, which could, in turn, manifest in the future as cardiac and/or cerebrovascular disorders, including cerebral small vessel disease (CSVD) and cognitive deficits.^14^

The most important neuroimaging features of CSVD include brain atrophy, white matter hyperintensities (WMH) and white matter microstructural integrity, assessed using diffusion-tensor imaging (DTI).^15^^,^^16^ Measures derived from DTI are sensitive to changes in microstructural integrity of white matter tracts associated with hypertension and CSVD and, consequently, with cognitive decline.^17^ Importantly, combining brain imaging with measures of cognitive performance, measured using neuropsychological cognitive tests, provides a more thorough understanding of brain health.^18^ CSVD commonly affects processing speed and executive function through disruption of frontosubcortical networks, whereas working memory and episodic or visual memory may also be affected.^15^^,^^19^

In the long term, new-onset HDP may increase the risk of cognitive decline in middle-aged females.^20^ However, the effect of new-onset HDP on long-term brain structural changes and cognitive decline remains poorly understood and warrants further investigation. Therefore, our main objective was to evaluate the association of new-onset HDP with brain atrophy and neurologic damage and long-term cognition, that is, cognitive health later in life in females using the neurocognitive battery of tests and imaging data associated with CSVD available from the UK Biobank cohort.

## Study design

### Study participants

This retrospective cohort included participants from the UK Biobank (https://www.ukbiobank.ac.uk/), a large, population-based, prospective cohort of 502,505 participants aged 40 to 69 years at the time of baseline assessment (2006–2010), recruited across 22 assessment centers in the United Kingdom.^21^ At the 2014 follow-up, participants from the original UK Biobank study population underwent cognitive function tests. This date refers to the beginning of the UK Biobank imaging assessment, during which cognitive tests were administered alongside brain magnetic resonance imaging (MRI).

### Diagnosis of new-onset HDP

Cases were females with a history of new-onset HDP, with gestational hypertension, with or without proteinuria and/or edema. Qualifying International Classification of Diseases (ICD) codes–O13 (gestational [pregnancy-induced] hypertension without significant proteinuria), O14 (preeclampsia), O15 (eclampsia), and O16 (unspecified maternal hypertension)–as well as self-reported data (field code: 1073) were used for the diagnosis of new-onset HDP. All participants were also asked to report whether they had ever received a diagnosis of gestational hypertension or preeclampsia from a healthcare professional at the recruitment visit.

### Covariates

We considered the following risk factors measured at baseline, given their association with cognitive decline: age at recruitment, age at first live delivery, alcohol consumption, sleep duration, smoking, education, high-density lipoprotein cholesterol, low-density lipoprotein cholesterol, diabetes, waist to hip ratio, and depression.

A history of diabetes was identified using hospital inpatient admission records and mortality register data. Blood lipids (high-density and low-density lipoprotein cholesterol) were obtained from biological samples, specifically randomly selected ethylenediaminetetraacetic acid plasma samples, using a high-throughput Nuclear Magnetic Resonance-based metabolic biomarker profiling platform. Finally, waist-to-hip ratio was defined as the ratio of waist circumference to hip circumference, both of which were measured using the Wessex nonstretchable sprung tape measurer.

Covariates were measured at the baseline assessment (2006–2010), whereas brain MRI and cognitive outcomes were obtained at the imaging assessment (2014 onwards).

### Ethics approval and consent to participate

UK Biobank obtained Research Tissue Bank approval from its governing research ethics committee, as recommended by the National Research Ethics Service. Permission to use the UK Biobank Resource was approved by the access subcommittee of the UK Biobank Board. The data were derived from the UK Biobank under Application 45551 and the study was approved by the institutional review board of McGill University Health Centre.

### Case–control matching

At the baseline UK Biobank assessment (2006–2010), participants self-reported their reproductive history, including the number of live births and age at first live birth. For the present analysis, we included participants who reported at least 1 live birth because age at first live birth and number of live births were used for case–control matching. These births occurred before UK Biobank recruitment. A live birth ensured that participants had carried a pregnancy long enough to be at risk for HDP. No information was available on fetal loss. We matched participants with new-onset HDP who had undergone MRI and cognitive function tests to randomly selected participants with normotensive pregnancy and MRI and cognitive function tests data. Cases and controls were matched 1:4 using their age at the time of first pregnancy as well as parity (ie, number of births).

## Outcomes

### Brain volumes

The macroscopic neuroanatomic parameters of interest to the present study were obtained from T1-weighted imaging and include gray matter volume (field ID 25005), white matter volume (field ID 25007), and volume of WMH (field ID 25781), which were normalized to participant head size.^22^

The MRI outcomes analyzed in this study were imaging-derived phenotypes generated through standardized UK Biobank acquisition and automated image-processing pipelines rather than subjective radiologist readings. Gray and white matter tissue volumes were derived using automated tissue segmentation; WMH volume was derived from T1-weighted and T2-FLAIR imaging using automated lesion segmentation; and diffusion MRI was processed to derive tract-based fractional anisotropy measures. The volumetric measures were provided in mm³ and were converted to mL.

### Microstructural integrity

Fractional anisotropy (FA) (Category ID 135), the most common metric derived from DTI, was used as an indicator of white matter microstructural integrity.^23^, ^24^, ^25^ FA represents the extent to which the diffusion of water is constrained in a specific direction, ranging from 0 (isotropic diffusion) to 1 (anisotropic diffusion). Although multiple factors can affect this metric, lower values of FA are thought to reflect poorer white matter microstructural integrity.^26^

### Cognitive function tests

Cognitive assessment in UK Biobank was performed through touchscreen questionnaires at baseline (2006–2010) and at follow-up visits in 2012, 2014, and 2019.^27^ At the 2014 follow-up, more quantitative tests of cognitive function evaluating cognitive domains known to be associated with small vessel disease and cognitive decline with age were added, including executive function (Towers Rearranging and Symbol Digit Substitution), processing speed (Reaction Time and Symbol Digit Substitution), visual declarative memory (measured with Pairs Matching), and working memory (Numeric Memory test).^28^ To assess differences in cognition between participants with new-onset HDP and normotensive pregnancy participants, we used cognitive function tests available in the UK Biobank that are specific to CSVD.^29^ The details of these tests are provided in Supplemental Table 1.

The UK Biobank cognitive assessments are brief, computerized measures designed for population-scale research rather than clinical diagnosis, and they assess cognitive domains relevant to vascular cognitive impairment and cognitive aging. These tests are computerized adaptations of established neuropsychological paradigms (digit span, symbol-digit substitution, Tower of London-type planning, and reaction-time tasks), and their validity has been evaluated against standard reference tests.^28^

### Analyses

All analyses in the present study were performed with SAS 9.4 for Windows (SAS Institute, Inc., Cary, NC). Thresholds for statistical significance were set at *P*<.05 (two-tailed).

We compared participants with new-onset HDP and normotensive pregnancy. To examine the characteristics of the study population, we conducted univariate analyses comparing females with new-onset HDP with those with normotensive pregnancy, respectively, using *t*-tests for continuous and χ^2^ tests for categorical variables.

For the morphologic brain structural MRI analysis, T1-weighted and DTI data from 2014 onwards follow-up were used. *t*-tests were used to compare brain volumes between hypertensive cases and normotensive controls. To investigate associations between gray matter volume, white matter volume, and volume of WMH between new-onset HDP cases and normotensive pregnancy controls, general linear regression models were used within each group. Beta coefficients and 95% confidence interval (CI) were presented using forest plots.

### Principal component analysis

For analysis of microstructural integrity (DTI), FA values from 27 white matter tracts (Figure 1) were reduced through principal components analysis (PCA). PCA is a data reduction technique that transforms the data into a smaller number of ”principal” or key components that retain the most important patterns.^30^ This technique has been previously used in analyzing DTI data.^31^ The most significant principal components were selected on the basis of having an eigenvalue >1, as well as scree plot inspection (elbow criterion). The eigenvalue matrix and scree plot from the PCA analyses are in the Supplemental Materials (Supplemental Figure 1, A). All values for the outcomes were standardized using z-score. Regression analysis was then performed with the principal components as outcomes.^31^

**Figure 1:**
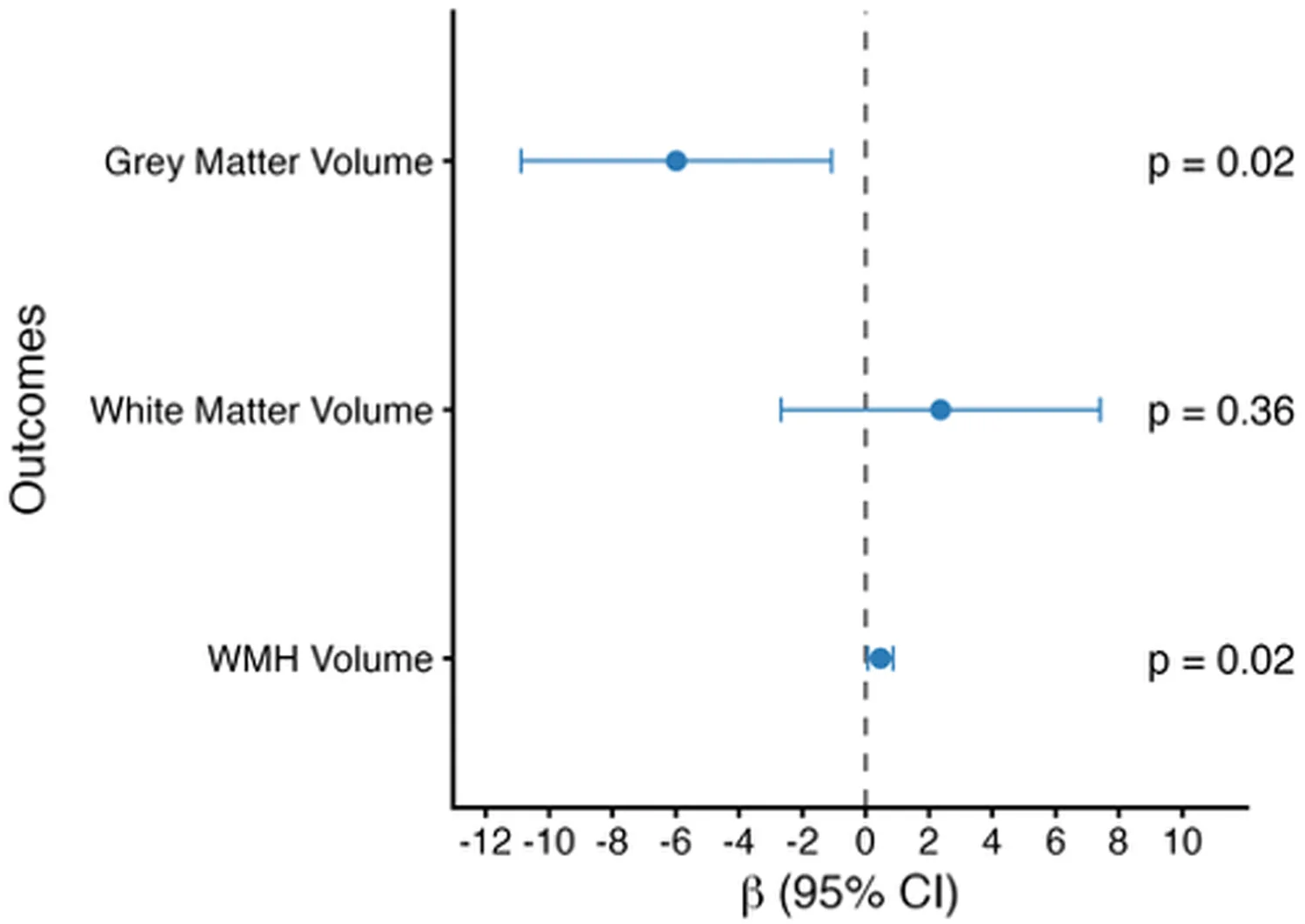
Differences in females with a history of new-onset hypertensive disorders of pregnancy versus females with normotensive pregnancy for morphologic brain structures, (mL)

Kaur. Long-term brain health among females with new-onset hypertensive disorders of pregnancy. Am J Obstet Gynecol Glob Rep 2026.

Similarly, values from 16 cognitive function tests related to CSVD and available in the UK Biobank were reduced through PCA, which has been previously used in analyzing cognitive function test data.^31^ The cognitive function tests that were loaded onto major principal components (PC factors) were then analyzed as outcomes (Supplemental Figure 2, A). To compare cognitive function outcomes (PC factors), *t*-tests were used to compare participants with new-onset HDP with those with normotensive pregnancy, and multivariable linear regression models were used while adjusting for the abovementioned covariates.

## Results

### Study population

Our study population included 273,227 females. We excluded females with no live births or missing data on the year of first live birth or missing brain MRI measurements (mean age ∼30 years postpartum). Next, we matched females with a history of new-onset HDP to those without HDP (1:4 case/control; 270 participants with new-onset HDP and 1080 without HDP) (Figure 2). Of the 270 participants with new-onset HDP, 148 (54.8%) had both an ICD code diagnosis and self-reported HDP, whereas 122 (45.2%) had an ICD-code diagnosis only; none had self-reported HDP without an ICD-coded diagnosis. The mean interval between baseline covariate assessment and MRI assessment was 9.25 (±2.02) years. Comparing the demographics and covariates at baseline (2006–2010), the mean age of females was 50±7 years. Females with new-onset HDP had a higher prevalence of vascular risk factors (Table), for instance, a higher prevalence of diabetes as well as gestational diabetes.

**Figure 2:**
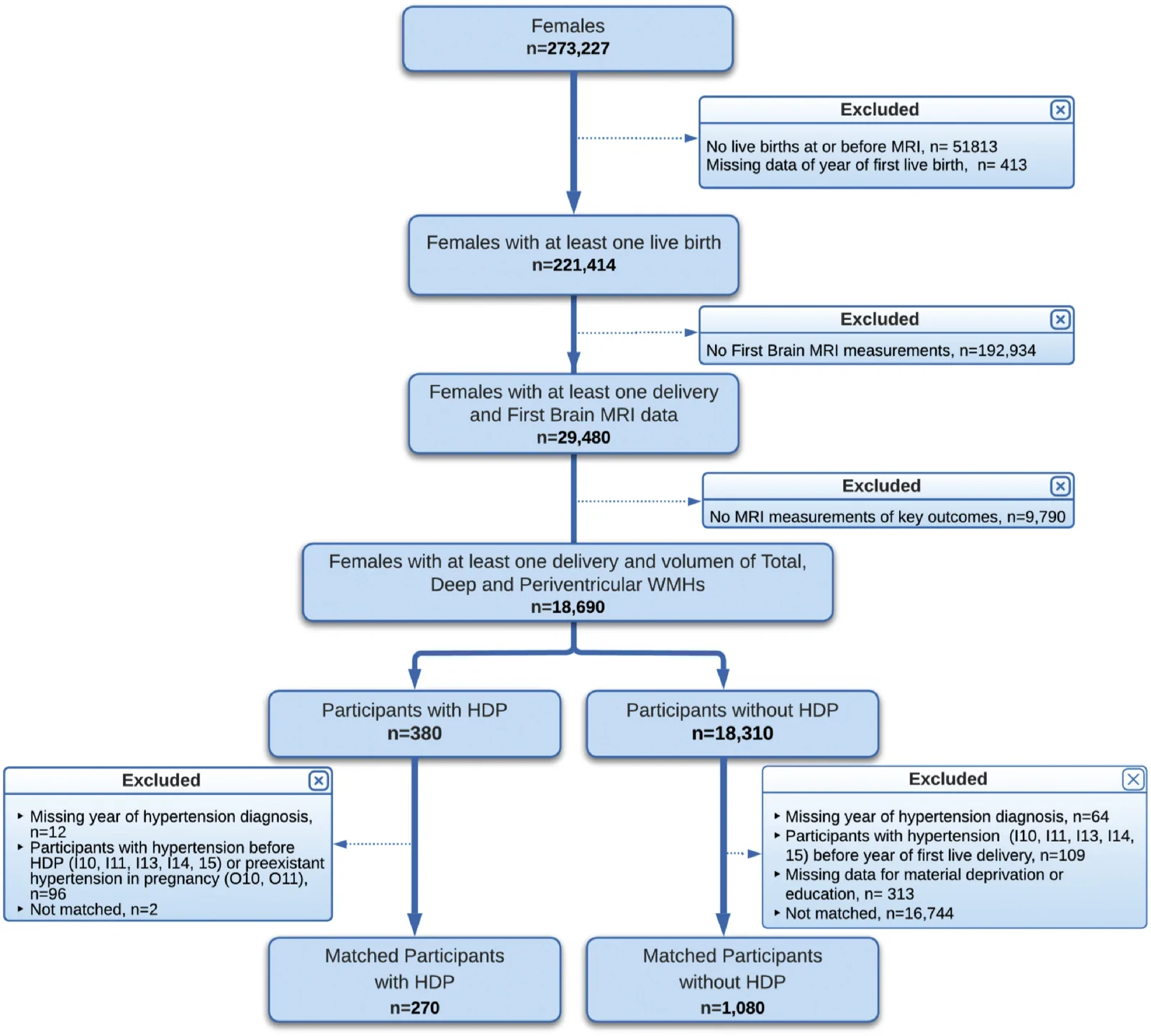
Flowchart of study participant selection

Kaur. Long-term brain health among females with new-onset hypertensive disorders of pregnancy. Am J Obstet Gynecol Glob Rep 2026.

**Table:** Characteristics of Study Population at Baseline

	HDP (n=270)	No HDP (n=1080)
Variable	Mean (SD)	Mean (SD)
Age at recruitment (y)	49.9 (7.4)	50.3 (6.9)
Number of births	2.2 (0.9)	2.2 (0.9)
Age at first birth (y)	29.9 (5.7)	29.7 (5.9)
Years from first delivery and MRI evaluation	29.6 (11.0)	30.3 (10.6)
Waist-to-hip ratio	0.8 (0.1)	0.8 (0.1)
	*n* (%)	*n* (%)
Diabetes	11 (4.1)	10 (0.9)
Gestational diabetes	8 (3.0)	0 (0.0)
Depression	18 (6.7)	83 (7.7)
Smoking	121 (44.8)	556 (51.5)
Short/long sleep duration	45 (16.7)	225 (20.8)
Daily alcohol consumption	44 (16.3)	185 (17.1)

*HDP*, hypertensive disorders of pregnancy; *MRI*, magnetic resonance imaging.

### Structural brain changes

Females with a history of new-onset HDP had lower gray matter volume (β= −6.0 mL; 95% CI, −10.9 to −1.1; *P*=.02) compared with females with normotensive pregnancy, as well as an increase in the volume of WMH (β=0.5 mL; 95% CI, 0.1–0.9; *P*=.02). There was no significant association for white matter volume (β=2.4 mL; 95% CI, −2.7 to 7.4; *P*=.36) (Figure 1).

### Changes in microstructural integrity with hypertension

#### Principal component analysis – factor selection

The 2 most significant principal components were selected as outcomes. Overall, the tracks that loaded onto the outcome factors were not isolated to a specific brain region but were distributed across the brain. The details of white matter tracts loadings to PC factors 1 and 2, that were used as outcomes, are detailed in supplemental data (Supplemental Figures 1, B and 1, C). The cognitive tests retained in the analysis included Numeric Memory, which assessed working memory; Symbol Digit Substitution and Reaction Time, which assessed executive function/processing speed; and Pairs Matching, which assessed visual declarative memory.

#### Multivariate linear regression

In females with a history of new-onset HDP, there was no association with white matter microstructural integrity for the FA outcome PC factor 1(β = −0.002; 95% CI, −0.005 to 0.002; *P*=.39) or PC factor 2 (β=0.002; 95% CI, −0.001 to 0.005; *P*=.20) when compared with females with normotensive pregnancy (Figure 3).

**Figure 3:**
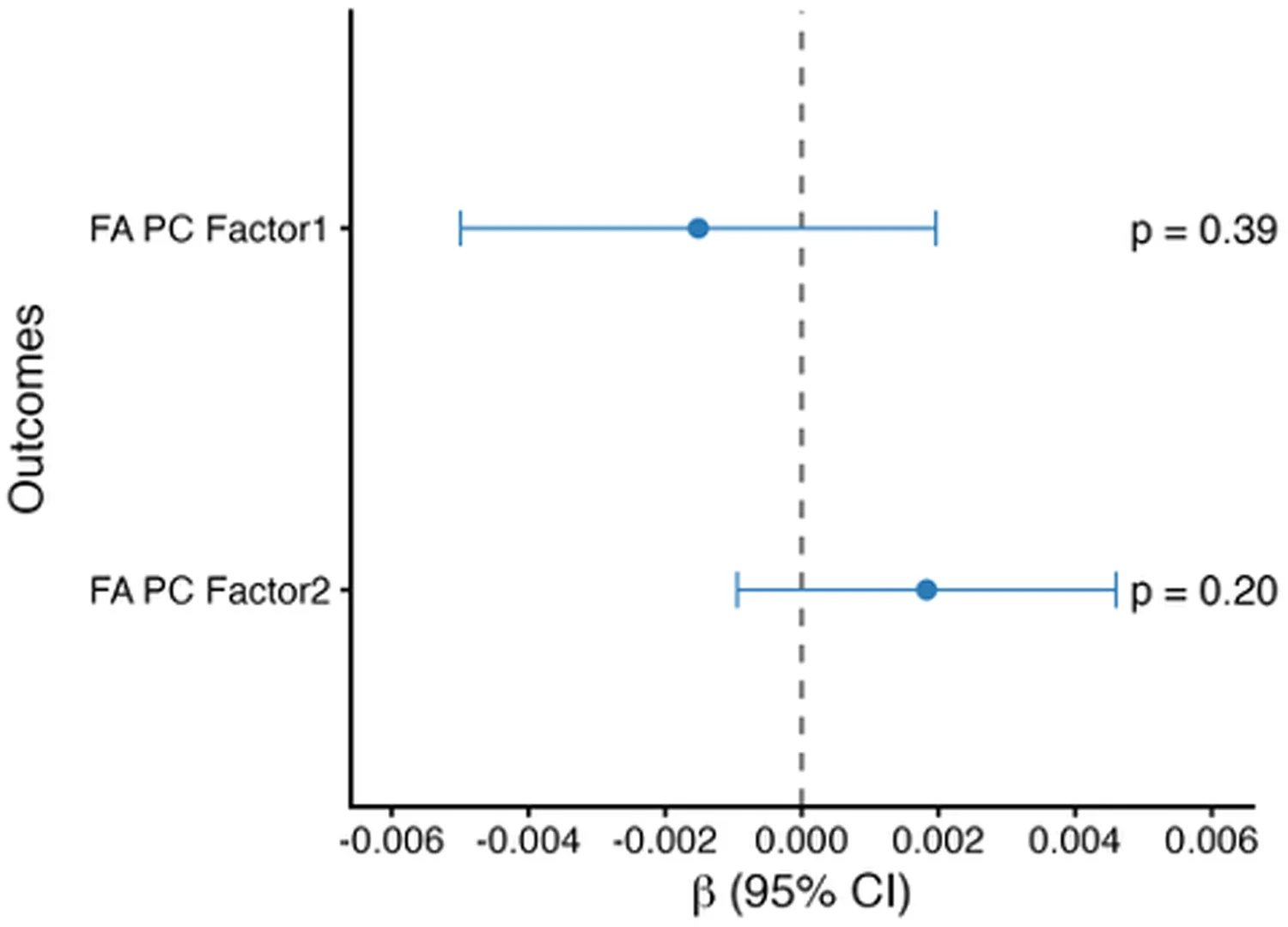
Differences in females with a history of new-onset hypertensive disorders of pregnancy versus females with normotensive pregnancy for fractional anisotropy (PC outcomes, z-score normalized). *PC*, principal component.

Kaur. Long-term brain health among females with new-onset hypertensive disorders of pregnancy. Am J Obstet Gynecol Glob Rep 2026.

### Cognitive function changes

#### Principal component analysis – factor selection

The 3 most significant principal components were selected as outcomes: PC factor 1 (working memory), PC factor 2 (executive function/processing speed), and PC factor 3 (visual declarative memory) (Supplemental Table 2). The details of cognitive function tests loading onto PC factors are detailed in the supplemental data (Supplemental Figures 2, B–D).

#### Multivariate linear regression

In females with a history of new-onset HDP, there was no association with cognitive function tests for PC factor 1, working memory (β= −0.3; 95% CI, −21.1 to 20.5; *P*=.98), factor 2, executive function or processing speed (β=1.5; 95% CI, −6.1 to 9.1; *P*=.69), and factor 3, visual declarative memory (β=−3.3; 95% CI, −12.8 to 6.2; *P*=.50) when compared with females with normotensive pregnancy (Figure 4).

**Figure 4:**
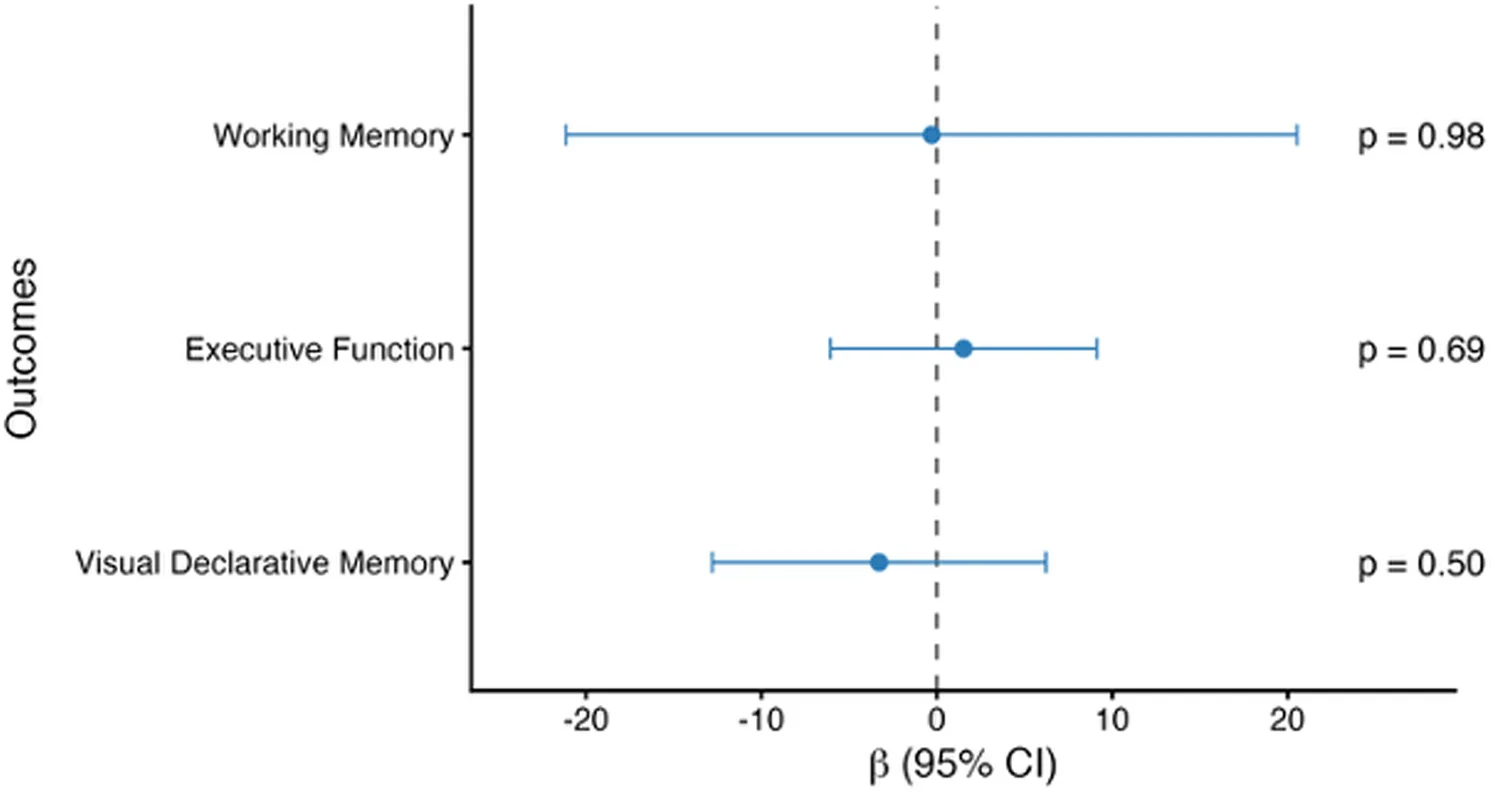
Differences in females with a history of new-onset hypertensive disorders of pregnancy versus females with normotensive pregnancy for cognitive function tests (PC outcomes). *New-onset HDP = gestational hypertension and preeclampsia. *HDP*, hypertensive disorders of pregnancy; *PC*, principal component.

Kaur. Long-term brain health among females with new-onset hypertensive disorders of pregnancy. Am J Obstet Gynecol Glob Rep 2026.

Overall, a history of new-onset HDP was associated with lower gray matter volume and greater volume of WMH later in life after pregnancy (Figure 1), whereas white matter volume (Figure 1), FA-derived white matter microstructural integrity (Figure 3), and cognitive outcomes (Figure 4) did not differ significantly between groups.

## Discussion

### Principal findings

In this study, we assessed the differences in brain health between females with and without new-onset HDP. We used multimodal MRI measures that included gray matter volume, white matter volume, and WMH volume, as well as DTI data (fractional anisotropy), which have been linked to CSVD, to examine structural and microstructural brain changes. Furthermore, we examined cognitive function tests related to CSVD.

Using data from the UK Biobank cohort, we evaluated both morphologic brain structural changes and microstructural integrity as well as cognitive performance in middle-aged and older females. We found that females with a history of new-onset HDP had lower gray matter volumes and greater WMH burden compared with those with normotensive pregnancies. Our study adds to the growing body of evidence that hypertension during pregnancy may be associated with long-term brain structural changes. However, we did not find any changes in cognitive performance in the present study.

### Results in the context of what is known

Our findings extend previous evidence from midlife cohorts examining HDP and brain health. In a study of the CARDIA cohort, Jiang et al^32^ reported that a history of gestational hypertension was associated with greater regional WMH burden during midlife. Although this study focused on cognitive outcomes and WMH, our study expands on these observations by examining brain morphology, white matter microstructural integrity, and cognition within a single cohort, providing a more comprehensive characterization of CSVD-related brain changes associated with HDP. These findings suggest that pregnancy-related hypertensive disorders may have enduring effects on cerebrovascular brain health that evolve across the life course and could lead to an increased risk of AD.

Previous studies have reported brain structural changes shortly after HDP and smaller brain volumes decades later.^20^^,^^33^^,^^34^ Our results are consistent with these findings, showing that these changes in morphologic brain structure are found even later in life with females with new-onset HDP exhibiting smaller gray matter volume years after giving birth than females without HDP. These findings highlight the impact of hypertension developed during pregnancy on poorer brain health later in life.

New-onset HDP has been associated with brain volume and structural changes after delivery.^33^ Females with a history of preeclampsia, a more severe form of HDP, have been reported to have lower cortical and subcortical gray matter volume soon after delivery compared with those with a normotensive pregnancy.^20^ Studies have also reported smaller brain volumes in females with new-onset HDP decades after pregnancy.^34^ Our findings align with previous studies that show that these changes in morphologic brain structure are found even later in life with females with new-onset HDP exhibiting smaller gray matter volume years after giving birth than females without HDP.

Interestingly, in spite of structural differences in reduced gray matter volume and a higher volume of WMH, we did not find any significant differences in cognitive performance. This could be attributed to the relatively younger age of females at the time of cognitive function tests. Birnie et al^35^ observed similar results, finding no evidence of a faster age-related cognitive decline in females with new-onset HDP as a whole (at a mean age of 51 years) compared with females with normotensive pregnancies. To evaluate whether these structural changes translate to cognitive decline or changes in white matter microstructure related to new-onset HDP later in life, a longitudinal study with long-term follow-up will be required.

Similarly, the lack of concordance in white matter tracts is consistent with previous research, which has shown that WMH and DTI metrics, including FA, capture related but distinct aspects of small vessel disease and do not always show parallel associations within the same population.^19^^,^^36^ WMH represents known, macroscopic tissue injury observable by MRI, whereas FA shows white matter microstructural features and does not directly correlate with lesion burden. Furthermore, WMH are spatially heterogeneous.^37^^,^^38^ Although FA may be altered within lesions and surrounding regions, tract-averaged measurements may reduce these localized effects.^19^^,^^36^^,^^39^ Therefore, the absence of FA differences in our study is more likely because of differences in the biology and measurement features of these imaging markers than a lack of underlying cerebrovascular abnormalities.

### Clinical implications

High blood pressure during pregnancy is a sex-specific factor that has been linked to brain structural changes and cognitive problems in females shortly after delivery.^40^^,^^41^ This increase in blood pressure during pregnancy may cause damage to the lining of blood vessels, which can continue throughout life, resulting in brain structural changes and a decrease in cognitive function.^40^^,^^41^

The increased WMH volume observed in the new-onset HDP group is consistent with prior research identifying WMH as a marker of small vessel disease. WMH are associated with aging and hypertension, and are an imaging biomarker of small vessel disease.^42^ Our results highlight that this association might be exacerbated in females with a history of new-onset HDP. Possible explanations for this association could be the effects of new-onset HDP on the brain, such as decreased arterial vessel dilatory capacity and impaired cerebrovascular regulation,^43^^,^^44^ making it more vulnerable to further damage later in life. Furthermore, females with a history of new-onset HDP have 3 to 4 times the likelihood of developing hypertension as they age compared with those with a normotensive pregnancy.^45^^,^^46^ The greater WMH volume suggests a long-term association between a history of new-onset HDP and brain structural changes.

Our findings show that a history of HDP was associated with lower gray matter volume and higher volume of WMH despite no significant cognitive changes. These findings show that HDP may affect brain health beyond pregnancy and that pregnancy history could be useful to inform cardiovascular and cerebrovascular risk assessment in later life. Clinically, this highlights the value of managing modifiable vascular risk factors, especially blood pressure level for later life brain health. Given the contribution of cerebrovascular disease to cognitive decline and dementia, these findings may also have implications for later AD risk.^47^

### Research implications

Future research should focus on identifying the underlying biological mechanisms that cause the association between new-onset HDP and CSVD-related morphologic brain structural changes and cognitive decline, as well as on the possibility of early detection and prevention measures. More research is also needed to understand the relationship between new-onset HDP and menopause in terms of CSVD progression in the longer term. Finally, more research is needed to understand the impact of social determinants of health or gender-related factors that could affect the association between new-onset HDP and brain health.

### Strengths and limitations

Our results highlight the significance of new-onset HDP as a female-specific risk factor for poorer brain health; nonetheless, this study has some limitations. The key limitation is the relatively young age of females at the time of MRI measurement and cognitive function tests, which may not have captured all aspects of the long-term impact of new-onset HDP on brain health through CSVD pathology. Moreover, the relatively small sample size could have reduced the statistical power of the study, potentially preventing the detection of more significant effects. In addition, although we adjusted for several clinical vascular risk factors, we were unable to adjust for social determinants of health, except education, that are known to have an impact on brain health. We also could not adjust for antihypertensive treatment because medication information recorded at a single time point would not capture treatment initiation, duration, adherence, intensity, medication changes, or cumulative blood pressure control over the approximately 30 years since pregnancy. Furthermore, hypertension, as well as covariates like smoking, diabetes, and alcohol consumption, which were self-reported, may be prone to reporting bias. The data set population is relatively healthy and educated, which may not reflect the general population.

Because the analysis was restricted to participants with at least one live birth, selection bias is possible. If more severe HDP is more likely to result in pregnancy loss and is also associated with greater later cerebrovascular injury, this restriction could attenuate the observed association; however, the overall direction and magnitude of this bias cannot be determined from the available data. Moreover, HDP ascertainment was from ICD code diagnoses and self-report in 54.8% of cases, rather than detailed obstetric medical-record adjudication. Therefore, some exposure misclassification is possible, and the direction and magnitude of any resulting bias cannot be determined from the available data.

Smoking and alcohol consumption were assessed at baseline. Because these behaviors may have changed over the approximately 9-year interval between baseline and the MRI and cognitive outcome assessment, baseline measurements may not fully represent behavior throughout follow-up, and some residual confounding is possible.

Nonetheless, we were able to see morphologic brain structural changes, with lower brain volume and a higher volume of WMH, in females with a history of new-onset HDP.

## Conclusion

This study emphasizes the importance of the impact of new-onset HDP on morphologic brain structure and cerebrovascular health through a proposed cerebral small vessel disease mechanism. We found that, when compared with age- and parity-matched females with normotensive pregnancy, females with new-onset HDP had lower gray matter volume and a higher volume of WMH.

Therefore, hypertension during pregnancy may represent a sex-specific risk factor for compromised brain health not only in the immediate postpartum period but also later in life. These findings highlight the significance of considering pregnancy history in long-term cardiovascular and cerebrovascular risk assessment and, thereby, the importance of addressing established modifiable vascular risk factors, particularly blood pressure level, to maintain brain health later in life.

## CRediT authorship contribution statement

**Amanpreet Kaur:** Writing – original draft, Visualization, Methodology, Funding acquisition, Formal analysis, Data curation, Conceptualization. **Adriana Angarita Fonseca:** Writing – review & editing, Methodology. **Hassan Behlouli:** Writing – review & editing, Validation, Formal analysis, Data curation. **M. Natasha Rajah:** Writing – review & editing, Supervision, Methodology. **Louise Pilote:** Writing – review & editing, Supervision, Methodology, Investigation, Funding acquisition, Conceptualization.

## Appendix Supplementary materials

Image, application 1

## Footnotes

Supplementary material associated with this article can be found in the online version at doi:10.1016/j.xagr.2026.100704.
