# Supplementary material for: Long-term brain health among females with new-onset hypertensive disorders of pregnancy
Source: AJOG Glob Rep. 2026 Oct 7;6(4):100704. doi: 10.1016/j.xagr.2026.100704 (PMC13757509; doi:10.1016/j.xagr.2026.100704)
Supplement: Supplementary file 1 [file mmc1.docx]

**Long-term Brain Health among Females with
New Onset Hypertensive Disorders of Pregnancy**

Amanpreet KAUR^1,2^ PhD, Adriana Angarita FONSECA^2^ PhD, Hassan BEHLOULI^2^ PhD,
M Natasha RAJAH^3,4^ PhD, and Louise PILOTE^1,2^ MD MPH PhD

1. Department of Medicine, Faculty of Medicine and Health Sciences, McGill University Health Centre, Montreal, Canada
2. Centre for Outcomes Research and Evaluation, Research Institute of the McGill University Health Centre, Montreal, Canada
3. Department of Psychology, Faculty of Arts, Toronto Metropolitan University
4. Department of Psychiatry, Faculty of Medicine and Health Sciences, McGill University, Montreal, Canada

Supplemental Table 1: Cognitive function tests that have been previously shown to be associated with SVD and are available in the UK Biobank

| Cognitive domain | Cognitive function test associated with SVD |
| --- | --- |
| Executive function | Trail making |
|  | Fluid intelligence |
|  | Matrix pattern completion |
|  | Symbol digit substitution |
|  | Tower rearrangement picture |
| Processing Speed | Reaction time |
| Memory | Numeric memory |
|  | Pairs matching |
|  | Prospective memory |

Supplemental Figure 1(a): Principal Component Analysis (PCA) Outcomes – Diffusion Tensor Imaging (Fractional Anisotropy)

|  | **Eigenvalue** | **Cumulative** |
| --- | --- | --- |
| **1** | 13.44 | 0.450 |
| **2** | 2.04 | 0.57 |
| **3** | 1.40 | 0.62 |

Supplemental Figure 1 (b) – PC Factor 1 – PC factor loading for Fractional Anisotropy

PC Factor1

|  | Factor1 |
| --- | --- |
| Forceps major | 0.19934 |
| Posterior thalamic radiation (L) | 0.15528 |
| Inferior fronto-occipital fasciculus (L) | 0.14096 |
| Inferior longitudinal fasciculus (L) | 0.13788 |
| Posterior thalamic radiation (R) | 0.13525 |
| Inferior fronto-occipital fasciculus (R) | 0.13164 |
| Inferior longitudinal fasciculus (R) | 0.1256 |
| Anterior thalamic radiation (R) | 0.11644 |
| Anterior thalamic radiation (L) | 0.11566 |
| Middle cerebellar peduncle | 0.08239 |
| Uncinate fasciculus (R) | 0.07918 |
| Acoustic radiation (L) | 0.0727 |
| Uncinate fasciculus (L) | 0.06951 |
| Acoustic radiation (R) | 0.06808 |
| Forceps minor | 0.02582 |
| Corticospinal tract (L) | -0.01329 |
| Corticospinal tract (R) | -0.02073 |
| Superior longitudinal fasciculus (L) | -0.03572 |

Supplemental Figure 1 (c) - PC Factor 2 – PC factor loading for Fractional Anisotropy

PC Factor2

|  | Factor2 |
| --- | --- |
| Superior thalamic radiation (R) | 0.42049 |
| Superior thalamic radiation (L) | 0.39682 |
| Corticospinal tract (L) | 0.21665 |
| Corticospinal tract (R) | 0.20373 |
| Superior longitudinal fasciculus (R) | 0.17557 |
| Superior longitudinal fasciculus (L) | 0.1369 |
| Forceps minor | 0.07543 |

Supplemental Figure 2 (a): Principal Component Analysis (PCA) Outcomes – Cognitive Function Tests

|  | **Eigenvalue** | **Cumulative** |
| --- | --- | --- |
| **1** | 3.92 | 0.22 |
| **2** | 2.44 | 0.35 |
| **3** | 1.65 | 0.44 |

Supplemental Figure 2 (b) – PC Factor 1 – PC factor loading for cognitive function tests

PC Factor 1

|  |  |
| --- | --- |
| Number of rounds of numeric memory test performed | 0.37892 |
| Maximum digits remembered correctly | 0.35545 |
| Time to complete test | 0.34997 |

Supplemental Figure 2 (c) – PC Factor 2 – PC factor loading for cognitive function tests

PC Factor 2

|  |  |
| --- | --- |
| Number of symbol digit matches made correctly | 0.45613 |
| Number of symbol digit matches attempted | 0.43631 |
| Mean time to correctly identify matches | -0.37703 |

Supplemental Figure 2 (d) – PC Factor 3 – PC factor loading for cognitive function tests

PC Factor 3

|  |  |
| --- | --- |
| Number of incorrect matches in round | 0.5787 |
| Time to complete round | 0.53091 |

Supplemental Table 2 – Cognitive Function Tests Selected as PC Outcomes

| Factor 1 | | |
| --- | --- | --- |
| Maximum digits remembered correctly | Numeric memory | Memory (working) |
| Number of rounds of numeric memory test performed |  |  |
| Time to complete test |  |  |
| Factor 2 | | |
| Mean time to correctly identify matches | Reaction time | Executive function/ Processing speed |
| Number of symbol digit matches attempted | Symbol digit substitution |  |
| Number of symbol digit matches made correctly |  |  |
| Factor 3 | | |
| Number of incorrect matches in round | Pairs matching  round 2 | Memory (visual declarative) |
| Time to complete round |  |  |
